# A Personalized Muscle–Tendon Assessment and Exercise Prescription Concept Reduces Muscle–Tendon Imbalances in Female Adolescent Athletes

**DOI:** 10.1186/s40798-025-00817-w

**Published:** 2025-02-07

**Authors:** Theresa Domroes, Kolja Weidlich, Sebastian Bohm, Falk Mersmann, Adamantios Arampatzis

**Affiliations:** 1 Department of Training and Movement Sciences, Humboldt-Universität Zu Berlin, Unter Den Linden 6, 10099 Berlin, Germany; 2 Berlin School of Movement Science, Berlin, Germany

**Keywords:** Muscle–tendon diagnostics, Tendon training, Female youth athletes, Tendon adaptation, Overuse, Individualization, Injury risk

## Abstract

**Background:**

Imbalances between muscle strength and tendon stiffness influence the mechanical demand on the tendon (i.e., tendon strain) and may increase tendon injury risk. The purpose of this study was to identify muscle–tendon imbalances and to promote a more balanced musculotendinous adaptation through a personalized assessment and exercise prescription concept in female adolescent handball athletes (13–16 years).

**Methods:**

At four measurement time points during a competitive season, we used dynamometry and ultrasonography to assess knee extensor muscle strength, patellar tendon stiffness and strain. Tendon micromorphology was assessed with a peak spatial frequency (PSF) analysis of proximal tendon ultrasound images. Muscle–tendon imbalances were identified based on tendon strain during maximum voluntary contractions. A control group (*n* = 15) followed their usual training. In the intervention group, athletes with a deficit in tendon stiffness (strain ≥ 9%; *n* = 6) or no muscle–tendon imbalances (strain between 4.5% and 9%; *n* = 15) performed exercises (3x/week for 32 weeks) with a personalized load to reach ~ 6.2% tendon strain to predominantly promote tendon or both muscle and tendon adaptation. Individuals with a muscle strength deficit (strain ≤ 4.5%; *n* = 1) trained with submaximal loads to failure to promote muscle strength.

**Results:**

In the intervention group we found lower fluctuations of maximum tendon strain (*p* = 0.005) and a decrease in tendon strain over time (*p* = 0.010), which was more pronounced in individuals with initially high tendon strain. While there were no systematic changes in muscle strength or tendon stiffness at the group level (*p* > 0.05), the marked decrease in tendon strain in individuals with a deficit in tendon stiffness was caused by a predominant increase in tendon stiffness. Overall, the prevalence of muscle–tendon imbalances was reduced in the intervention group, while it temporarily increased in the control group. PSF did not differ between groups at baseline but decreased significantly in the intervention group (*p* = 0.013).

**Conclusions:**

The findings suggest that the personalized concept is suitable to promote a more uniform adaptation of knee extensor muscle strength and patellar tendon stiffness and to reduce the prevalence of musculotendinous imbalances in female adolescent athletes, which may have important implications for tendon injury prevention.

**Trial Registration:**

DRKS, DRKS00035110. Registered 20 September 2024–retrospectively registered, https://drks.de/search/de/trial/DRKS00035110.

## Background

Imbalances in the relationship of muscle strength and tendon stiffness have been frequently observed in athletic populations of different age groups from childhood [1] through adolescence [2–5] to adulthood [4, 6, 7]. If muscle strength and tendon stiffness do not adapt to a similar extent, this will affect tendon operating strain during muscle contractions. The deformation of tendons during movement enables the storage and return of elastic strain energy [8, 9] and further influences the muscle’s working conditions and, accordingly, its capacity to generate force and produce power [10–12]. Thus, it is conceivable that imbalances between muscle strength and tendon stiffness, causing either high or low levels of tendon strain, may impair an effective muscle–tendon interaction [13, 14], which may influence movement efficiency and performance. Further, evidence suggests that ultimate tendon strain is quite constant across tendons and species [15], so that the magnitude of strain can be used as an indicator of the mechanical demand placed on the tendon. Accordingly, a muscle–tendon imbalance due to a deficit in tendon stiffness relative to muscle strength leads to increased tendon strain during maximum effort muscle contractions [2, 4] and increases the mechanical demand on the tendon tissue, which may increase tendon injury risk. Though the etiology of tendon injuries, such as tendinopathy, is certainly multifactorial [16, 17], tendon overuse is considered one of the key factors for the development of tendon pain and associated functional impairments [16, 18]. In vitro experimental data have provided evidence that initial tendon strain serves as a predictor of time or cycles to failure during static and cyclic loading [19] and that high strain levels of 9% induce net catabolic processes in the tendon tissue [20]. In line with this, in vivo findings of our group indicate a possible association of increased tendon strain during maximum voluntary contractions and a disorganization of the tendon’s structural appearance in male adolescent athletes [21]. Further, we recently found in a prospective longitudinal study, that adolescent athletes who develop tendon pain show higher tendon strain levels prior to the onset of pain compared to athletes that remain asymptomatic. Thereby, the risk for developing tendon pain was 2.3-fold higher in individuals whose tendon strain exceeded 9% during maximum effort muscle contractions [22]. Though these findings need to be further substantiated to develop a clear understanding of the relation between tendon strain, structural degeneration and tendon pain in vivo, the data indicate that the repeated exposure to increased tendon strain may increase tendon injury risk. Consequently, targeted exercises to counteract muscle–tendon imbalances in athletes may contribute to tendon injury prevention and may possibly promote a more effective interaction of the muscle–tendon unit [13, 14]. At the individual level, the direction of muscle–tendon imbalances (i.e., a relative deficit in tendon stiffness or muscle strength) as well as their severity can differ considerably between athletes [23]. Therefore, exercises to effectively reduce muscle–tendon imbalances may require an individual assessment of muscle–tendon properties. Based on this, the decision to specifically promote either tendon stiffness or muscle strength and the respective loads during exercises could be personalized [23].

One of the underlying factors in the development of muscle–tendon imbalances is the different responsiveness of muscle and tendon to certain stimuli [24, 25]. While muscles respond to a wide range of exercise intensities, including mechanical as well as metabolic stress [26, 27], tendon strain seems to be an important factor for tendon adaptation [24, 25, 28, 29]. Further, plyometric loading, which is quite prevalent in sports such as handball, seems suitable to increase muscle strength [30], while its effects on the tendon are less clear [29, 31, 32]. These discrepancies may lead to a non-uniform adaptation of muscle strength and tendon stiffness, which in turn leads to higher fluctuations in tendon strain over time. On the other hand, these differences can be used to select exercise modalities that specifically target tendon or muscle adaptation to prevent or reduce musculotendinous imbalances [23]. For the adaptation of tendon mechanical properties, tendon strain is a key factor [20, 33, 34]. Previous systematic intervention studies on the Achilles tendon have shown that tendon strain magnitudes between 4.5 and 6.5% repeatedly applied with a loading duration of about 3 s were particularly effective in increasing tendon stiffness compared to lower strain magnitudes [24, 28, 29]. To accurately reach this effective tendon strain region during exercises, load prescriptions based on maximum voluntary contraction (MVC) or one-repetition maximum alone may be insufficient as tendon strain at a given relative force level can differ considerably between individuals, especially in populations with a high prevalence of muscle–tendon imbalances [23, 35]. Accordingly, a training prescription based on tendon strain may be more effective in targeting tendon adaptation. Further, if strain during exercises is controlled, potentially harmful high-level tendon strain during training can be avoided. The muscle, on the other hand, responds to a variety of mechanical and metabolic stimuli [26, 27], whereas it was recently shown that the tendon is less sensitive to exercise-induced metabolic stress [25]. Consequently, exercises that induce insufficient strain in the tendon but high metabolic stress in the muscle may be used to specifically promote muscle adaptation. Based on these considerations, we developed a theoretical concept for a personalized muscle and tendon assessment and exercise prescription [23] by using maximum tendon strain during MVCs to identify musculotendinous imbalances and to subsequently select the most effective training modality to either restore or preserve a balanced relationship between muscle and tendon. We could recently show that this concept proved suitable to counteract muscle–tendon imbalances in male adolescent [36] as well as male adult [7] athletes. It is, however, not clear to date if this also applies to female athletes.

While the occurrence of muscle–tendon imbalances in female athletes is still barely investigated, recent findings of our group provide first evidence that the prevalence does not seem to differ between male and female adolescent athletes [5], which underlines the necessity of measures to counteract musculotendinous imbalances in both these sexes. Regarding sex differences in the adaptation of muscle and tendon to training, there also exists only limited data. There are indications that relative muscle strength and mass adaptations in response to resistance training are rather similar between male and female adults [37] and adolescents [38]. For tendon adaptation, some evidence suggests an attenuated response to mechanical loading in women compared to men [39] as well as sex-specific differences in training-induced increases in tendon stiffness [40]. On the other hand, even though sex-specific differences were not specifically addressed, we found that targeted exercises for the Achilles tendon with a strain magnitude of ~4.6% were effective in inducing tendon stiffness adaptations in a mixed sample with mainly female participants [24].

Therefore, the current study investigated how the implementation of a personalized muscle–tendon assessment and exercise prescription concept into the regular training of female adolescent athletes affects the adaptation of knee extensor muscle strength and patellar tendon stiffness as well as the prevalence of musculotendinous imbalances. Based on the available literature [7, 36], we hypothesized that the personalized exercise prescription promotes a more balanced adaptation of muscle strength and tendon stiffness, resulting in reduced fluctuations of maximum patellar tendon strain over time.

## Methods

### Participants and Experimental Design

A group of female adolescent elite handball athletes (aged 13–16 years) was recruited for the present study. We used a two-seasons prospective design to evaluate the personalized concept with the first season serving as control period and the second as intervention period. The necessary sample size was calculated in a power analysis (G*Power, version 3.1.9.7). Due to the lack of data on training-induced tendon adaptation in adolescent girls, we based the power analysis on pre-post changes in tendon stiffness after a non-personalized tendon exercise intervention in male adolescent athletes, where we observed a large effect (d = 1.4; [3]). For a power of 0.9, we calculated a sample size of 8 per group. Considering a potential drop-out as well as possibly smaller effects of exercise on tendon stiffness in females [39], we recruited 18 athletes from the representative team of the local handball association for a control group, which received only their sport-specific training. In the second season, 25 athletes from the same team (similar age group; including 12 girls that had already participated in the control group) were recruited for an intervention group which integrated exercises according to the personalized muscle–tendon assessment and exercise prescription concept into their regular training. All participants had a weekly training duration of at least 9 h (approx. 60% handball specific training, 40% strength and athletic training; excluding competition) and were experienced with systematic resistance training (> 1 year of experience). Exclusion criteria were any neurological or musculoskeletal impairments of the lower extremities. Participants with patellar tendon pain were included if maximum voluntary muscle strength testing was possible. All participants and their legal guardians gave written informed consent to the experimental procedures, which were approved by the ethics committee of the Humboldt-Universität zu Berlin (ref. no. HU-KSBF-EK_2020_0005) and performed in accordance with the standards of ethics outlined in the Declaration of Helsinki. The study was retrospectively registered at the German Clinical Trial Register (DRKS; trial registration number: DRKS00035110).

We assessed knee extensor muscle strength, patellar tendon mechanical properties and micromorphology as well as the prevalence of tendon pain and pain-related disability in both groups at four measurement time points during a competitive season (M1, M2 and M3: in-season; M4: transition period). Due to restrictions in the scheduling of the measurements (e.g., holidays, training camps, tournaments), the exact time span between measurements differed between groups and individuals with on average 11, 13 and 13 weeks between measurements in the control group and 11, 12 and 11 weeks in the intervention group. The training intervention started 2 weeks after M1 resulting in an average intervention duration of 32 weeks. All measurements were performed on the dominant leg, defined as the leg used to kick a ball. As an estimate for biological maturity we calculated the years to peak height velocity (PHV) using age and height according to the redeveloped prediction equation suggested by Moore and colleagues [41]. Patellar tendon pain and pain-related disability were assessed at every measurement time point with the validated German version of the VISA-P questionnaire [42] considering the symptoms of the past 2 weeks. Participants were categorized as symptomatic with a score ≤ 87 points, which represents the minimum clinically important difference [42] from the maximum score of 100 points.

In the control group, three participants were excluded from the study due to movement artifacts in the ultrasound recordings of the tendon, which did not allow an accurate analysis of tendon properties, and three participants did not take part in M4 due to undisclosed reasons, leading to a total of 57 observations. In the intervention group, two participants were excluded from the study due to sickness/injury not related to the study and one due to leaving the handball team. One participant did not take part in M2 due to sickness and one participant in M3 as well as four participants in M4 were excluded due to injuries not related to the study. Further, data of one participant in M2 and one in M3 were excluded due to movement artifacts in the ultrasound recordings and one in M3 due to missing data, resulting in a total of 79 observations in the intervention group.

### Personalized Muscle–Tendon Assessment and Exercise Prescription Concept

**Fig. 1:**
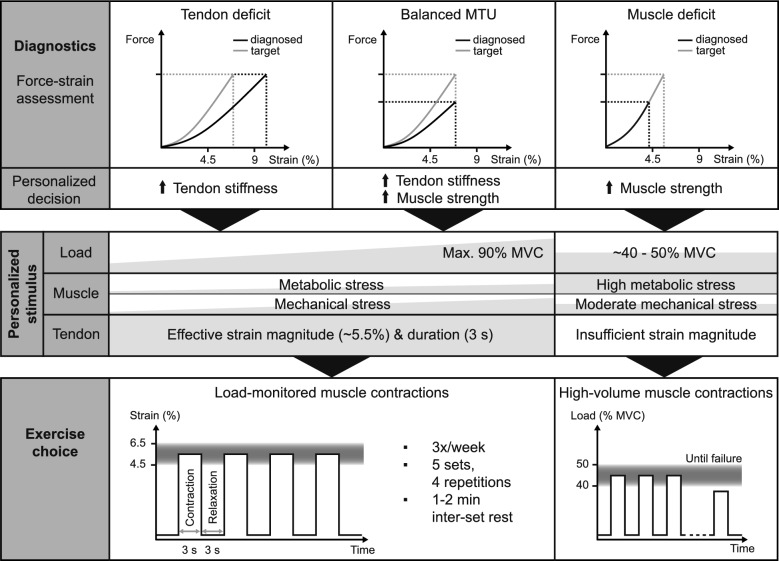
Personalized muscle–tendon assessment and exercise prescription concept. Based on individual muscle–tendon diagnostics, a tendon stiffness deficit (tendon strain ≥ 9%), a balanced muscle–tendon unit (MTU; tendon strain between 4.5 and 9%) or a muscle strength deficit (tendon strain ≤ 4.5%) were identified. In individuals with a tendon stiffness deficit, the target was to decrease tendon strain by increasing tendon stiffness, while the target for individuals with a balanced MTU was to increase both muscle strength and tendon stiffness. For both groups, the training load was personalized in order to reach an effective strain magnitude for tendon adaptation (4.5–6.5%), whereby the relative training load was limited to 90% MVC. With this training design, the load relative to the MVC decreases with increasing maximum tendon strain, so that the stimulus for muscle adaptation is lower in individuals with high maximum tendon strain (i.e., individuals with a tendon stiffness deficit). In participants with a muscle strength deficit, the target was to increase tendon strain through increases in muscle strength. The respective personalized stimulus included high-volume muscle contractions at a moderate load (~ 40–50% of a maximum voluntary contraction (MVC)) until failure to cause a sufficient metabolic stimulus for muscle adaptation, while the tendon strain magnitude should be too low to induce substantial tendon adaptation

Based on the results of their muscle–tendon diagnostics, the participants of the intervention group included personalized exercises into their regular training, which were designed to promote a more balanced adaptation of muscle and tendon (Fig. 1) [23]. We used maximum tendon strain during MVCs as a marker for musculotendinous imbalances [23]. If maximum patellar tendon strain was below 4.5%, we assumed a deficit in muscle strength compared to tendon stiffness, so that the intervention exercises in these individuals aimed at primarily increasing muscle strength. Accordingly, the “muscle training” consisted of 4 sets of dynamic knee extension contractions to failure with a load that allowed 25 to 30 repetitions per set, as this should cause a metabolic stimulus that is sufficient to trigger muscle adaptational processes [26, 27] while the mechanical stimulus on the tendon is considered too low to induce substantial tendon adaptation [24]. In individuals with maximum tendon strain between 4.5 and 9% we assumed a rather balanced relationship between muscle strength and tendon stiffness, while strain magnitudes ≥ 9% were considered indicative for a deficit in tendon stiffness compared to muscle strength. Even though it is unlikely from a physiological perspective that there exists a clear threshold of tendon strain that can have potentially harmful consequences for the tissue, high-level tendon strain of 9% or higher proved suitable to identify athletes with an increased risk to develop tendon pain [22]. Therefore, this threshold was used for the current assessment and exercise prescription concept. In both participants without muscle–tendon imbalances and individuals with a relative deficit in tendon stiffness, the intervention exercises consisted of five sets of four fixed-end knee extension contractions with a contraction duration of 3 s according to the most effective loading protocol for the tendon from our earlier systematic research [24, 28, 29]. Thereby, training load was personalized to provide an evidence-based effective strain magnitude (i.e., 4.5–6.5% tendon strain) [24, 28] for tendon adaptation. Consequently, we determined the relative force needed to induce 5.5% tendon strain for each participant based on their maximum patellar tendon strain during MVCs under the simplified assumption of a linear relationship between tendon force and strain. The maximum relative training load was limited to 90% of maximum force to ensure an accurate execution of the training protocol. This means that participants who would, according to our calculation, need a training intensity above 90% MVC to reach 5.5% tendon strain (i.e., participants with maximum tendon strain ≤ 6.1%), all trained at 90% of their MVC. With this training design, the load relative to the MVC decreases with increasing maximum tendon strain, so that it can be assumed that the stimulus for muscle adaptation is lower in individuals with high maximum tendon strain (i.e., individuals with deficits in tendon stiffness), thus inducing primarily tendon adaptation. The higher load relative to the MVC in individuals with low maximum tendon strain (i.e., individuals with balanced muscle strength and tendon stiffness) on the other hand is expected to provide an adequate stimulus for both muscle and tendon adaptation [24, 28].

The exercises were performed in a mobile training device [36] equipped with a digital scale to monitor the training load. From an MVC performed in the training device and the prescribed relative loading intensity (i.e., % MVC), the absolute load for the exercises was then determined for each participant individually. The participants were instructed to complete the personalized exercises three times per week and to train both legs with the same relative load. To account for strength adaptations during the training period, a new MVC was recorded in the training device every 2 weeks for the fixed-end tendon exercises. The MVCs were usually conducted after a short warm-up at the beginning of the regular resistance/athletic training session into which the interventional exercises were integrated. The load during “muscle training” was increased as soon as more than 30 repetitions were achieved in one set. With regards to possible changes in tendon mechanical properties, we additionally determined maximum tendon strain (during three fixed-end ramp contractions) at an interval of 5 to 6 weeks between the regular measurement time points to adjust the prescribed training type or load accordingly.

### Assessment of Knee Extensor Muscle Strength

To determine knee extensor muscle strength, we assessed the maximum knee joint moment during fixed-end maximum voluntary contractions. After a self-selected warm up (including submaximal and 5 maximal jumps), the participants were seated on a custom-built mobile measurement system [5, 36] with a hip joint angle of about 90° (0° corresponds to supine position) and strapped to the seat with a non-elastic belt. A strap was fixed around the shank of the dominant leg just above the ankles and was attached to a force sensor (2 kN; Biovision, Wehrheim, Germany) in series with a non-elastic rope, which was adjusted so that the force sensor was aligned in the direction of force application perpendicular to the shank and that the knee joint angle reached 60° during contractions (0° represents full knee extension; measured with a goniometer). This knee joint angle was chosen, as it is approximately the optimal angle for maximum knee extensor force generation [43]. After completing ten submaximal fixed-end knee extension contractions with increasing effort as additional warm-up, familiarization and preconditioning of the tendon, the participants performed two MVCs with 2–3 min rest between trials. Using a custom-written MATLAB interface (version R2016a), force data was recorded at 200 Hz and filtered with a second-order Butterworth filter with a cut-off frequency of 6 Hz. A moving average filter with a window size of 50 ms was used to determine maximum force values. The knee joint moment was calculated by multiplying the recorded force with the lever arm of the applied force (i.e., distance from the lateral epicondyle to the middle of the strap around the shank) and was then corrected for moments of gravity using the data provided by Dempster [44] for the estimation of the mass and center of mass of the foot and shank. The MVC-trial with the highest maximum moment was used for further analysis.

### Assessment of Patellar Tendon Mechanical Properties

To assess patellar tendon mechanical properties, we determined the tendon’s force–elongation relationship during five fixed-end ramp contractions in the same setup used for the MVCs. The participants received visual feedback to steadily increase effort from rest to maximum within 5 s. The forces acting on the tendon were determined by dividing the knee joint moment by the patellar tendon moment arm which was estimated based on anthropometry [2] and adjusted to the knee joint angle with the data provided by Herzog and Read [45]. Maximum tendon force is thereby defined as the force calculated from the highest MVC trial of each participant. Tendon elongation during the ramp contractions was recorded with a 10 cm ultrasound probe (ultrasound system: MyLab60; Esaote, Genova, Italy; probe: LA923, 7.5 MHz; 25 Hz image frequency), which was fixed over the longitudinal axis of the patellar tendon with a modified knee brace. Ultrasound and force data were synchronized with a manually released 5 Volt trigger signal. Tendon elongation was digitized by tracking the displacement of the deep insertion of the tendon at the patellar apex and the tibial tuberosity in the ultrasound images with a semi-automatic tracking software (Tracker Video Analysis and Modeling Tool V. 5.1.5; Open Source Physics, Aptos, CA, United States). Tendon rest length was determined from the ultrasound images prior to the onset of the contraction as the length of the slack tendon in a relaxed state (i.e., at 60° knee joint angle). It was calculated with a spline fit through the deep insertion marks and two additional points along the border of the tendon and averaged for all five trials. The force–elongation relationship for each single ramp trial was saved as piecewise-linear polynomial function in MATLAB (version R2019b; “interp1” function with “pp” option), using all the measured data of the respective ramp as input. The five trials were then averaged to achieve a high reliability and observer independence [46]. Therefore, at each measured data point, all single ramp trials were evaluated using their respective piecewise-linear polynomials and then averaged (using the “fncmb” function in MATLAB). The resulting mean piecewise-linear polynomial was evaluated up to 80% of maximum tendon force, as this was the highest relative force all participants were capable of achieving during the ramp contractions. Finally, a second-order polynomial fit passing through zero was applied to the averaged force–elongation data points. Patellar tendon stiffness was calculated as the quotient between 50 and 80% of maximum tendon force from this polynomial function. To consider individual differences in patellar tendon rest length and their effect on patellar tendon stiffness, we further normalized patellar tendon stiffness by multiplying it with patellar tendon rest length. Normalized tendon stiffness describes accordingly the slope of the tendon force-strain relationship. Maximum patellar tendon strain was calculated by extrapolating tendon elongation to the maximum tendon force based on the second-order polynomial fit of the force–elongation curve and dividing it by patellar tendon rest length. To ensure an adequate transferability of the strain values determined in the experimental setup and the strain achieved during tendon exercises, the training device was designed to closely resemble the experimental setup and was individually adjusted so that the knee joint angle reached 60° during contractions [36]. As both MVCs, in the measurement system and training device, were performed at the same knee joint angle, we assumed similar forces on the tendon and accordingly similar tendon strain.

### Assessment of Tendon Micromorphology

A peak spatial frequency analysis of ultrasound recordings of the proximal part of the patellar tendon was conducted to assess tendon micromorphology. The assessment took place prior to the measurement of muscle strength and tendon mechanical properties to minimize the potential influence of acute loading on tendon microstructural appearance. The participants were positioned supine with their knee flexed to 90° (measured with a goniometer). This angle was chosen as tendon slack is commonly removed in this angle, while tendon strain induced by passive joint forces is still low [21]. An ultrasound probe (ultrasound system: MyLab60; Esaote, Genova, Italy; probe: linear array LA523, 13 MHz, depth: 3.0 cm) was placed over the patellar tendon parallel to its longitudinal axis below the most distal apex of the patella and two short sequences were captured. The images were then analyzed with a custom-written MATLAB interface (version R2016a; MathWorks, Natick, MA, United States). A polygonal region of interest (ROI) was defined that spanned 40% of the tendon’s rest length (measured as described above) covering the full thickness of the tendon from the deep insertion on the patella to the tendon midportion. Using the approach suggested by Bashford and colleagues [47], all 32 × 32 pixel kernels contained within the ROI were analyzed by applying a 2D Fast Fourier transform and a high-pass filter with a radial frequency response and half-power cutoff frequency of 1.23 mm^−1^. To increase frequency resolution, the filtered kernels were zero padded in both directions to a size of 128 × 128 pixels. The distance of the peak spatial frequency (PSF) to the spectral origin in the frequency spectrum of all analyzed kernels was averaged. The mean PSF of the two recorded trials was then used for further analysis. A more isotropic speckle pattern in the ultrasound images results accordingly in lower PSF values which indicate a lower packing density and more disorganized alignment of the collagen bundles as has been observed tendinopathic tendons [47, 48]. At the patellar tendon PSF values of 1.4 to 1.8 mm^−1^ (interquartile range) have been reported for athletes with tendinopathy in comparison with values between 1.7 and 2.0 mm^−1^ for healthy controls [48].

### Statistics

We formulated a linear mixed-effects model using the *nlme* package in RStudio (version 4.1.2; RStudio, PBC, Boston, MA, United States) with group-specific y-intercept, slope and variance of the residuals to analyze time- and group-dependent changes. An advantage of linear mixed models is that they can handle missing data and are robust against violations of the normality assumption [49], which was not given for age, stiffness, normalized stiffness, and maximum tendon elongation according to the Shapiro–Wilk test applied to the normalized residuals. The model equation was

1 $${\text{y}}_{{{\text{ij}}}} = \, \beta_{0} + \, \beta_{{1}} {\text{t}}_{{{\text{ij}}}} + {\text{ g}}_{{\text{i}}} \beta_{{2}} + {\text{ g}}_{{\text{i}}} \beta_{{3}} {\text{t}}_{{{\text{ij}}}} + {\text{ b}}_{{0{\text{i}}}} + {\text{ b}}_{{{\text{1i}}}} {\text{t}}_{{{\text{ij}}}} + {\text{ r}}_{{{\text{ij}}}}$$

where i is the index for participant, j is the index for the measurement session, g_i_ = 0 in the control group and 1 in the intervention group, t_ij_ is the time point (in weeks) for the measurement session j of the participant i, β_0_ is the y-intercept constant for the control group, β_1_ is the slope constant for the control group, β_2_ is the y-intercept constant for the difference between control and intervention group, β_3_ is the slope constant for the difference between control and intervention group, b_0i_ is the participant-specific y-intercept (random effect), b_1i_ is the participant-specific slope (random effect), and r_ij_ is the residual. Consequently, the terms including g equal zero in the control group, so that β_0_ and β_1_ represent the respective constants for intercept and slope. In the intervention group, β_0_ + β_2_ and β_1_ + β_3_ represent the constants for intercept and slope, respectively. Accordingly, we tested the significance of changes over time in the control group (slope constant β_1_), baseline differences between groups (intercept constant β_2_), and group differences in time-dependent changes (slope constant β_3_; hereafter referred to as time by group interaction). We further tested separately for the intervention group if the slope differed significantly from zero. Note that age was only tested for baseline differences. Individual and group differences in the time span between measurements were accounted for by coding the time points of the measurements in weeks for each participant individually.

Effect sizes (Cohen’s d) were calculated using the means and the pooled standard deviation derived from the model equation for group differences at baseline and for differences between M1 and M4 in both groups separately. The inclusion of random effects in the applied model considers individual differences in intercept and slope, so that the residuals of the model can be used as a measure of individual fluctuations from a linear development. Therefore, we determined the estimated standard deviations of the residuals of the linear mixed model for the contextually relevant parameters. To assess whether the standard deviation of the residuals differed between groups, we formulated two linear mixed models according to Eq. 1, with one model assuming homogeneity of the residual variance (no group differences) and the other model allowing for group differences in the residual variance. The two models were then compared by a likelihood ratio test, from which it can be inferred whether the standard deviation of the residuals differed between groups. The correlation of maximum tendon strain at M1 and relative changes in tendon strain from M1 to M4 was analyzed for both groups separately using the Pearson correlation coefficient (r) to investigate whether there exists a relationship between initial tendon strain and changes in tendon strain over time. The significance level for all statistical tests was set to α = 0.05.

## Results

Anthropometric data are shown in Table 1. The intervention group was significantly older than the control group (*p* = 0.004) with a greater biological maturity (estimated as offset from PHV; *p* = 0.044). There were no baseline differences in body height (*p* = 0.837) and mass (*p* = 0.735) between groups. Biological maturity, body height and mass increased significantly over time in the control (*p* < 0.001, *p* < 0.001, and *p* = 0.002) and intervention group (*p* < 0.001, *p* = 0.021, and *p* = 0.014) with no time by group interaction (*p* = 0.072, *p* = 0.057, and *p* = 0.468). Average VISA-P scores and the number of symptomatic athletes (VISA-P score ≤ 87) are depicted in Table 1. The percentage of symptomatic athletes during the measurement period ranged from 0 to 40% in the control group and 0 to 13.6% in the intervention group. In the intervention group, only one participant had a maximum tendon strain during MVCs ≤ 4.5% at M1–M3 and accordingly performed the “muscle training” during the intervention period. All other participants without muscle–tendon imbalances (*n* = 15 at baseline) or a relative deficit in tendon stiffness (*n* = 6 at baseline) performed the personalized tendon exercises. Here, the personalized exercise load relative to the individual MVC was on average (mean ± standard deviation) 70.5% ± 13.1%, 76.2% ± 11.6%, and 74.8% ± 10.1% for M1, M2, and M3 respectively, with an overall range from 52.4 to 90%. This corresponded to a tendon operating strain during training of 6.5% ± 0.8%, 5.9% ± 0.6%, and 6.1% ± 0.6% (mean for all measurement time points: 6.2%), with an overall range from 4.3 to 7.7% based on the experimentally determined force–elongation relationship.

**Table 1:** Anthropometric data, average VISA-P scores and number of symptomatic athletes

	Control				Intervention			
	M1 (*n* = 15)	M2 (*n* = 15)	M3 (*n* = 15)	M4 (*n* = 12)	M1 (*n* = 22)	M2 (*n* = 20)	M3 (*n* = 19)	M4 (*n* = 18)
Age (years)^*^	14.3 ± 0.7	14.5 ± 0.7	14.7 ± 0.7	15.0 ± 0.8	15.1 ± 0.8	15.2 ± 0.8	15.4 ± 0.7	15.7 ± 0.8
Maturity offset (years)^*,†, §^	2.47 ± 0.80	2.68 ± 0.79	2.87 ± 0.79	3.11 ± 0.81	3.01 ± 0.71	3.12 ± 0.75	3.23 ± 0.64	3.47 ± 0.77
Body height (cm)^†, §^	169 ± 8	170 ± 8	170 ± 7	171 ± 7	168 ± 6	169 ± 5	168 ± 6	169 ± 6
Body mass (kg)^†, §^	63.1 ± 9.8	63.9 ± 10.2	64.7 ± 10.5	66.0 ± 10.1	62.2 ± 8.0	62.5 ± 8.0	62.3 ± 7.2	63.2 ± 9.4
VISA-P score	91.1 ± 12.0	98.2 ± 4.8	97.5 ± 7.0	97.9 ± 7.2	97.1 ± 7.6	100 ± 0	97.8 ± 9.6	100 ± 0
No. of sympt. athletes	6 (40.0%)	2 (13.3%)	2 (13.3%)	1 (8.0%)	3 (13.6%)	0 (0.0%)	1 (5.3%)	0 (0.0%)

The values are mean ± standard deviation of the given experimental data. Maturity offset refers to the estimated years to peak height velocity. Symptomatic was defined as VISA-P score ≤ 87

^*^Significant baseline difference between groups, ^†^ significant change over time in the control group, ^§^ significant change over time in the intervention group (*p* < 0.05); there was no time by group interaction (*p* > 0.05). Note that age was only tested for baseline differences between groups

**Fig. 2:**
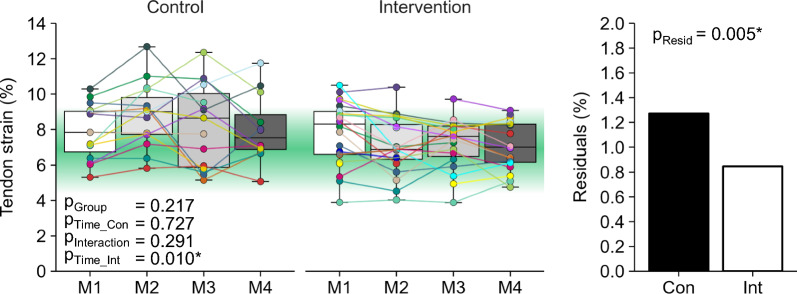
Maximum patellar tendon strain for both groups and each measurement time point (M1–4) during the season. The given experimental data is shown in box plots with individual data depicted in colors. The green area marks the strain range that indicates a balanced muscle-tendon unit (i.e., tendon strain between 4.5% and 9%). The estimated standard deviation of the residuals of the linear mixed model as a measure of parameter fluctuations over time is shown in the column on the right. The *p* values for baseline differences between groups (p_Group_), changes over time in the control group (p_Time_Con_), time by group interaction (p_Interaction_), changes over time in the intervention group (p_Time_Int_) as well as group differences of the standard deviation of the residuals (p_Resid_) are given. * marks significant *p* values (< 0.05)

**Fig. 3:**
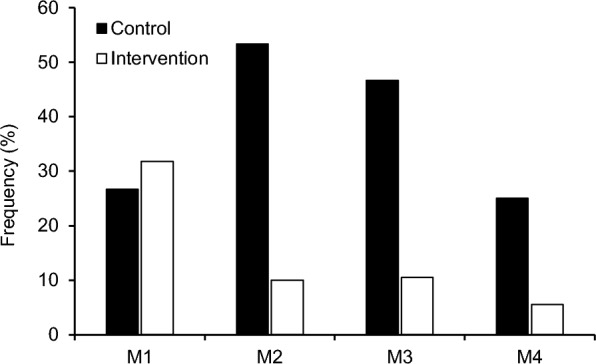
Frequency of athletes demonstrating muscle–tendon imbalances (i.e., tendon strain ≤ 4.5 or ≥ 9%) for the control (black) and intervention group (white) in the four measurement sessions (M1–4) over the season

Maximum tendon strain during MVCs did not differ significantly between the control and intervention group at baseline (*p* = 0.217, d = 0.40; Fig. 2). We observed a significant decrease in maximum tendon strain over time in the intervention group (*p* = 0.010, d =  − 0.45), but no significant changes in maximum tendon strain in the control group (*p* = 0.727, d = −0.10) and no time by group interaction (*p* = 0.291). The intervention group demonstrated lower fluctuations (residuals) of patellar tendon strain (*p* = 0.005; Fig. 2) and descriptively a lower frequency of athletes with low- or high-level patellar tendon strain (i.e., strain ≤ 4.5% or ≥ 9%) compared to the control group (14% vs. 38%; Fig. 3).

**Fig. 4:**
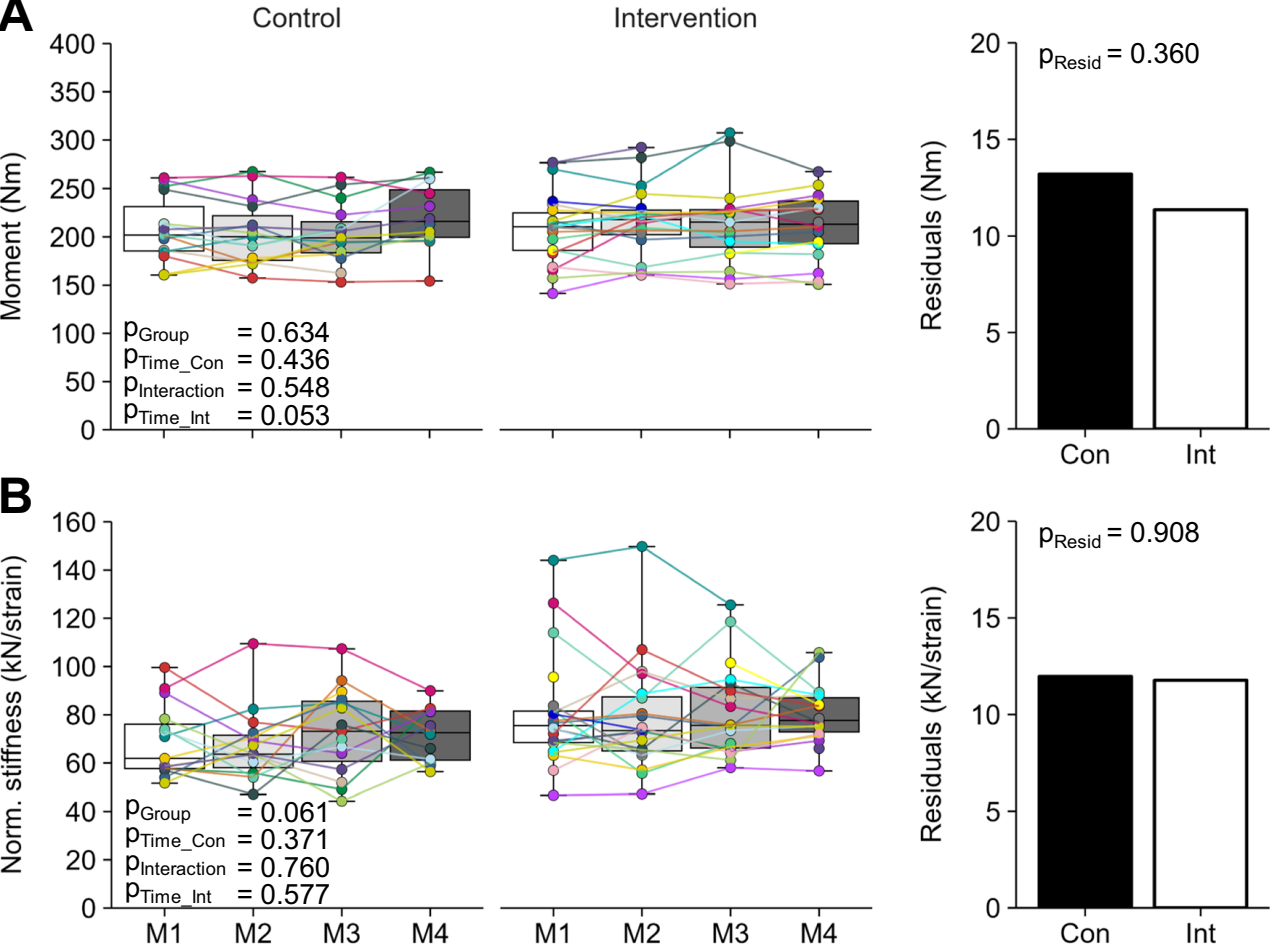
**A** Maximum knee joint extension moment and **B** normalized patellar tendon stiffness for both groups and each measurement time point (M1–4) over the season. The given experimental data is shown in box plots with individual data depicted in colors. The estimated standard deviation of the residuals of the linear mixed model as a measure of parameter fluctuations over time is shown in the column on the right. The *p* values for baseline differences between groups (p_Group_), changes over time in the control group (p_Time_Con_), time by group interactions (p_Interaction_), changes over time in the intervention group (p_Time_Int_) as well as group differences of the standard deviation of the residuals (p_Resid_) are given. * marks significant *p* values (< 0.05)

We found no significant differences in maximum knee joint moment between groups at baseline (*p* = 0.634, d =  − 0.16; Fig. 4A) as well as no group differences in knee joint moment normalized to body mass (*p* = 0.483, d =  − 0.23; Table 2) and force applied to the tendon (*p* = 0.589, d =  − 0.18; Table 2). Neither maximum knee joint moment, nor normalized knee joint moment or tendon force changed significantly over time in the control (*p* = 0.436, d = 0.15; *p* = 0.938, d =  − 0.02; *p* = 0.585, d = 0.11) or intervention group (*p* = 0.053, d = 0.30; *p* = 0.243, d = 0.26; *p* = 0.075, d = 0.30) and there was no time by group interaction in all three parameters (*p* = 0.548, *p* = 0.425, and *p* = 0.490). The fluctuations of maximum knee joint moment, normalized knee joint moment and tendon force were not significantly different between groups (*p* = 0.360, *p* = 0.476, and *p* = 0.385). There were no significant differences between groups in tendon stiffness (*p* = 0.071, *d* =  − 0.58; Table 2) and normalized tendon stiffness (*p* = 0.062, d =  − 0.60; Fig. 4B) at baseline. Both parameters did not change significantly over time in the control group (*p* = 0.512, d = 0.18 and *p* = 0.371, d = 0.23) or intervention group (*p* = 0.789, d = 0.07 and *p* = 0.577, d = 0.13) and there was no time by group interaction (*p* = 0.761 and *p* = 0.760) as well as no group differences in fluctuations (*p* = 0.875 and *p* = 0.908).

**Table 2:** Maximum normalized knee joint moment, patellar tendon force, stiffness, maximum elongation, rest length and PSF

	Control				Intervention			
	M1 (*n* = 15)	M2 (*n* = 15)	M3 (*n* = 15)	M4 (*n* = 12)	M1 (*n* = 22)	M2 (*n* = 20)	M3 (*n* = 19)	M4 (*n* = 18)
Norm. moment (Nm/kg)	3.34 ± 0.48	3.24 ± 0.50	3.19 ± 0.56	3.40 ± 0.64	3.36 ± 0.37	3.46 ± 0.40	3.44 ± 0.56	3.38 ± 0.49
Tendon force (N)	3939 ± 593	3840 ± 597	3819 ± 566	4127 ± 620	3951 ± 614	4079 ± 613	4048 ± 736	4019 ± 630
Tendon stiffness (N/mm)	1486 ± 378	1446 ± 299	1544 ± 319	1501 ± 246	1697 ± 461	1650 ± 427	1710 ± 361	1687 ± 281
Max. tendon elongation (mm)^§^	3.7 ± 0.9	4.1 ± 1.0	3.9 ± 1.1	3.8 ± 0.8	3.7 ± 0.9	3.4 ± 0.9	3.4 ± 0.7	3.4 ± 0.7
Tendon rest length (mm)^§^	47.0 ± 4.6	46.8 ± 4.7	47.3 ± 4.9	48.0 ± 4.1	47.3 ± 4.6	47.5 ± 4.7	47.4 ± 4.7	47.8 ± 4.4
PSF (mm^−1^)^‡, §^	1.78 ± 0.14	1.79 ± 0.10	1.83 ± 0.12	1.79 ± 0.12	1.83 ± 0.14	1.76 ± 0.13	1.76 ± 0.14	1.73 ± 0.19

All values are mean ± standard deviation of the given experimental data. PSF: Peak spatial frequency at the proximal patellar tendon

^‡^Significant time by group interaction, ^§^ significant change over time in the intervention group (*p* < 0.05)

Patellar tendon rest length (Table 2) was not significantly different between groups at baseline (*p* = 0.801, d =  − 0.09) and did not change over time in the control group (*p* = 0.716, d = 0.02). There was no significant time by group interaction in patellar tendon rest length (*p* = 0.090), but a significant increase in the intervention group (*p* < 0.001, d = 0.14). Fluctuations of patellar tendon rest length were significantly higher in the control group (*p* < 0.001). Maximum tendon elongation during MVCs (Table 2) did not differ significantly between groups (*p* = 0.294, d = 0.34) and showed no changes in the control group (*p* = 0.681, d =  − 0.11) as well as no time by group interaction (*p* = 0.372). There was a significant decrease in maximum tendon elongation in the intervention group (*p* = 0.020, d =  − 0.39) and the fluctuations of maximum tendon elongation were significantly lower in the intervention group (*p* = 0.013). PSF at the proximal patellar tendon was not significantly different between groups at baseline (*p* = 0.403, d = −0.25; Table 2) and did not show any systematic changes over time in the control group (*p* = 0.700, d = 0.14). There was, however, a significant time by group interaction (*p* = 0.041) and a significant decrease in PSF in the intervention group (*p* = 0.013, d = −0.89). Fluctuations of PSF did not differ significantly between groups (*p* = 0.217).

**Fig. 5:**
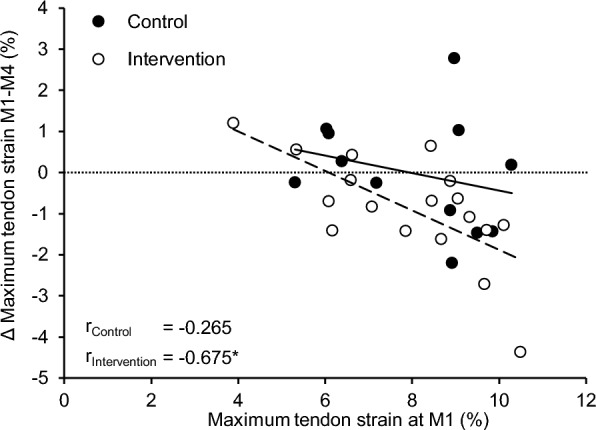
Association of maximum patellar tendon strain at the first measurement time point (M1) and changes of maximum patellar tendon from the first to fourth measurement time point (M1 to M4) for the control (black, solid line) and intervention group (white, dashed line). r Pearson correlation coefficient; * significant association (*p* < 0.05)

Lastly, we found a significant negative correlation between maximum tendon strain at M1 and changes in maximum tendon strain from M1 to M4 in the intervention group (r =  − 0.675, *p* = 0.002) but not in the control group (r =  − 0.265, *p* = 0.404; Fig. 5).

## Discussion

In the present longitudinal study the effects of a personalized muscle–tendon assessment and exercise prescription concept on imbalances between knee extensor muscle strength and patellar tendon mechanical properties were investigated during a competitive season in female adolescent elite handball athletes. The concept is based on using maximum tendon strain to identify muscle–tendon imbalances and to provide individualized exercise recommendations to counteract these. Accordingly, we individually assessed the athletes’ tendon strain during MVCs. Participants with maximum tendon strain ≤ 4.5% were categorized as having a relative deficit in muscle strength, individuals with tendon strain between 4.5% and 9% were defined as having no muscle–tendon imbalances, and individuals with maximum tendon strain ≥ 9% were categorized as having a relative deficit in tendon stiffness. In the intervention group, athletes with a muscle strength deficit (*n* = 1 at baseline) were assigned exercises with a moderate intensity until failure to specifically promote muscle strength. Athletes with no muscle tendon imbalances (*n* = 15 at baseline) or a relative deficit in tendon stiffness (*n* = 6 at baseline) were assigned tendon exercises with a load that was personalized based on their individual maximum tendon strain in order to reach strain values between 4.5% and 6.5% during training, which is considered an effective stimulus for tendon mechanical and morphological adaptation [24, 28, 29]. As hypothesized, fluctuations of maximum patellar tendon strain during MVCs over time were lower in the intervention group compared to the control group. In addition, we observed a decrease in maximum patellar tendon strain during the season only in the intervention group and found a reduction in the frequency of athletes with muscle–tendon imbalances (i.e., tendon strain ≤ 4.5% or ≥ 9%) from 32% at M1 to 6% at M4 in the intervention group, while the prevalence of muscle–tendon imbalances in the control group temporarily increased during the season. These results show that the concept proved suitable to specifically modulate maximum patellar tendon strain through a personalized regulation of tendon stiffness or muscle strength and accordingly reduce muscle–tendon imbalances in female adolescent athletes.

The relation between the force acting on a tendon and the tendon’s normalized stiffness determines in vivo tendon strain. Thereby, high fluctuations of tendon strain over time can be indicative for an imbalanced adaptation of muscle and tendon, which can lead to highly increased or decreased tendon strain. Accordingly, previous studies did not only find greater fluctuations of tendon strain during MVCs in athletes compared to non-athletic populations [1, 2, 6], but also provided evidence for a high prevalence of high-level tendon strain in male adult [4, 7] as well as male and female adolescent athletes [2, 4, 5]. In line with this, tendon strain fluctuated greatly in the current control group, and all but one athlete with musculotendinous imbalances in both groups had a maximum tendon strain ≥ 9% indicative for a relative deficit in tendon stiffness. As ultimate tendon strain seems to be quite constant [15], strain can serve as an indicator for the mechanical demand placed on the tendon. Regarding the consequences of an increased mechanical demand through high-level tendon strain, our group has provided in vivo evidence that the risk of developing tendon pain is 2.3-fold greater in adolescent athletes with tendon strain during MVCs of 9% or higher [22] and that increased tendon strain may be associated with tendon structural degeneration [21]. These findings are supported by in vitro studies evidencing an increased injury risk for the tendon with repetitive loading with high tendon strain [19, 20]. Thereby, Wang and colleagues conclude that tissue damage and degenerative processes seem to exceed tendon repair mechanisms at long term exposure to strains as high as 9% [20]. Though the exact relationship between tendon strain and tendon tissue damage is not entirely clear in vivo, this suggests that high-level tendon strain may be a critical factor in the development of overuse-related tendon injuries. The successful reduction of tendon strain fluctuations and the reduced prevalence of high-level tendon strain in the present study are well in line with a recent study on male adolescent athletes, where we also observed lower fluctuations and a reduction of high-level tendon strain in response to the same personalized muscle–tendon assessment and exercise prescription concept [36]. Taken together, this indicates that the proposed personalized concept promotes a more uniform adaptation of muscle and tendon and may be used for tendon injury prevention in adolescent athletes independent of sex.

**Fig. 6:**
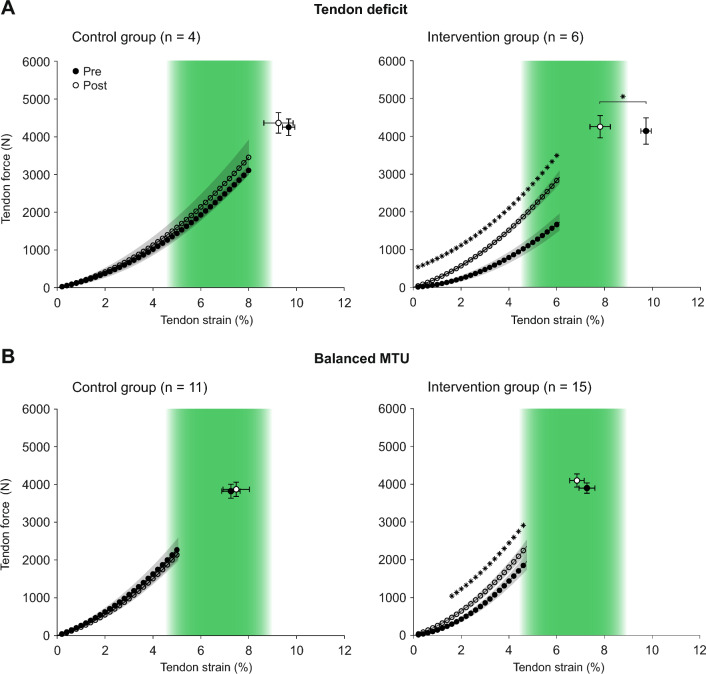
Patellar tendon force-strain relationship before (black) and after (white) the intervention period for (**A**) participants with a relative deficit in tendon stiffness at baseline (i.e., tendon strain ≥ 9%) and (**B**) a balanced muscle-tendon unit (MTU; tendon strain between 4.5% and 9%) at baseline in the control (left) and intervention group (right). The green area marks the strain range that indicates a balanced MTU. Data are depicted as mean ± standard error. * Significant differences between measurements in tendon force for given tendon strain or maximum tendon strain, respectively (p < 0.05)

From a theoretical point of view, in vivo tendon strain during MVCs can only change if either the force acting on the tendon or normalized tendon stiffness change. The training concept therefore aims at reducing tendon strain in individuals with high-level tendon strain through predominant increases in tendon stiffness. Towards lower maximum tendon strain, the stimulus for muscle adaptation increases due to the higher relative force during exercises according to the concept so that both muscle strength and tendon stiffness should increase and tendon strain remains rather unchanged. In individuals with low-level tendon strain, on the other hand, increases in muscle strength should predominate in order to increase tendon strain. In line with this, we found in the intervention group that participants with initially high tendon strain showed a greater reduction in tendon strain over time (Fig. 5). On the other hand, in the one participant with low-level tendon strain (i.e., ≤ 4.5%) at baseline, tendon strain increased slightly. Regarding the underlying mechanisms of these changes, the data of the participant with low-level tendon strain suggest that the increase in tendon strain was rather caused by a decrease in tendon stiffness, while muscle strength remained largely unchanged over time. Since this is based on one participant only, it is, however, difficult to draw any conclusions about the effectiveness of the applied training in participants with low-level strain. To better illustrate the effect of the intervention in participants with high-level tendon strain or no muscle–tendon imbalances at baseline, we separately plotted the force-strain relationships for the first and last measurement time point for these groups (Fig. 6). Additionally, we used a student’s t-test to assess significant differences in tendon force between measurement time points at 0.2% intervals of tendon strain as well as pre-post differences between maximum tendon strain and force. While we found no differences between M1 and M4 in the control group, participants in the intervention group with a tendon deficit showed a significant increase in tendon force needed to induce a given strain magnitude over the whole length of the force-strain relationship, which evidences an increase in normalized tendon stiffness. As maximum tendon force did not change over time, maximum tendon strain was significantly reduced in the intervention group (Fig. 6a). Together, these findings confirm the mechanisms proposed in the concept, that tendon strain at a given force is reduced in this group by an exercise-induced increase in stiffness. In the participants of the intervention group without muscle–tendon imbalances, we observed significant differences in tendon force for 1.6% tendon strain and above, but no significant changes in maximum tendon force or strain (Fig. 6b). While this indicates that the intervention caused small changes in tendon stiffness in this group, muscle strength was, contrary to the theoretical concept, unaffected. The results also differ somewhat from previous findings in male adolescent athletes, where tendon stiffness as well as muscle strength systematically increased in response to the same personalized tendon exercises [36]. It seems that the suggested loading modalities in the group with balanced muscle–tendon properties were not sufficient to introduce muscle strength improvements in the female athletes, which may indicate sex specific differences in the responsiveness of the quadriceps muscles to the applied training load. Although additional studies with systematic modulations of the exercise load are needed to confirm this, it needs to be pointed out that the personalized intervention seemed equally effective in reducing tendon strain fluctuations as well as muscle–tendon imbalances in both male [36] and female athletes.

If tendon strain is chronically increased, this may eventually lead to a mechanical overload of the tendon tissue which causes disruptions in its structural integrity [20, 50]. Using a PSF analysis of ultrasound images to quantify the tendon’s microstructural organization, it has been shown in vivo that tendinopathic tendons are characterized by greater collagen disorganization (i.e., lower PSF values) [47, 48]. Further, a lower structural integrity of the proximal patellar tendon has also been observed in asymptomatic athletes with increased tendon strain during MVCs and may point towards early degenerative processes in the tendon [21]. Accordingly, it is rather surprising that we found a decrease in PSF in the intervention group. Considering the low number of symptomatic athletes as well as the decreased prevalence of high-level tendon strain over time in the intervention group, it seems unlikely that the decrease in PSF represents a pathological consequence of tendon overload, especially since the average overall training volume and content did also not differ considerably between groups. Further, it needs to be pointed out that the mean PSF values for each measurement time point of the intervention group are well within the interquartile range reported for other asymptomatic athletic populations [48]. This suggests that the reduction in PSF may merely reflect physiological variation rather than a shift into the pathological range. One explanation for the variation in PSF may be that transient changes in tendon structural organization may occur within 2 days after vigorous physical activity [51]. Due to the high frequency of training and competition in the current groups as well as school-related constraints in the scheduling of the measurements, we could not control the time interval between physical activity and measurements. Though it was avoided to schedule measurements on the same day as sports activities, measurements often took place the day after training or competition. This may add to the variability of the data and complicates a clear interpretation of the findings.

To effectively monitor muscle–tendon imbalances over a longer period of time, reliable and accurate measurements of muscle strength and tendon stiffness are required. The reliability of the assessment of patellar tendon elongation has been investigated in a previous study from our group [46] demonstrating that the inclusion of five trials leads to a high reliability (≥ 0.95). Hence, we averaged five trials for each participant and each measurement time point in the current study. Since we used the same ultrasound device and a comparable analysis routine, we are quite confident that we were able to achieve a similarly high reliability in our study. Regarding the reliability of muscle strength measurements in children and adolescents, a meta-analysis [52] reported high intraclass correlations from 0.84 to 0.90 indicating that isokinetic dynamometry can be reliably used for muscle strength assessment in younger age groups. In addition, we made sure to properly familiarize participants with the MVC assessment by performing at least ten submaximal fixed-end contractions with increasing effort in the measurement set up. It also needs to be mentioned that all athletes in the current study were highly trained and had at least one year experience with systematic resistance training, so that we expect that neither the measurements nor the training intervention were significantly affected by differences in the participants’ ability to perform the given exercises. Another aspect that may influence muscle and tendon assessment and training in female athletes are hormonal fluctuations with the menstrual cycle. Though still insufficiently investigated, especially in adolescents, the current literature suggests that cycle-related hormonal variations do not seem to affect tendon properties in adult women [53–56]. Regarding the influence of the menstrual cycle on muscle strength, the findings in the current literature are also inconclusive. While some studies report an influence, others did not find any effects or report contradictory results regarding how the different cycle phases affect muscle strength [57]. Taken together, these results suggest that the menstrual cycle has little influence on the investigated parameters. Further, since the measurements were scheduled without factoring in the menstrual cycle phase, the cycle phase at the measurements was quasi randomized across and within participants and the interventional exercises were performed across all cycle phases, so that we would not expect any systematic influence on the data.

For the accurate assessment of muscle–tendon imbalances, maximum tendon strain needs to be determined. Because of the inability of the participants to reach their MVC force levels during slow ramped contractions, we extrapolated tendon elongation between 80 and 100% MVC. Due to the averaging of five trials, the assessment of the force–elongation relationship was quite robust, and the R^2^ for the second-order polynomial that was used to extrapolate tendon elongation was on average > 0.98 in every measurement time point. Additionally, running the statistical analysis with tendon strain measured at 80% MVC did not affect the main findings, so that we can assume that our results were not considerably affected by the extrapolation.

Another aspect that warrants further discussion is the training adherence of the participants in the intervention group. After familiarizing the athletes and coaches with the interventional exercises, the exercises were implemented into the regular training and mostly conducted autonomously. Except for holidays, training camps and other events preventing regular training, the exercises were scheduled three times per week. While this was the only feasible way to realize such a long-term intervention in this group, we had to rely on the reports given by the coaches regarding general training adherence and cannot make any clear statements about differences in individual training participation that may have influenced our results. Further, it needs to be mentioned that the participants in the intervention group were on average older and biologically more mature compared to the control group. When running our analysis with a subset of the intervention group age-matched to the control group, we obtained, however, similar statistical results. Therefore, we are quite confident that the small age difference did not affect our main findings.

## Conclusions

In conclusion, this longitudinal study evaluated a personalized muscle–tendon assessment and exercise prescription concept, in which we used maximum patellar tendon strain to identify muscle–tendon imbalances and to prescribe personalized exercises as a countermeasure. We found that the concept proved suitable to promote a more uniform adaptation of knee extensor muscle strength and patellar tendon stiffness and to reduce the prevalence of musculotendinous imbalances in female adolescent athletes. Thereby, we observed that most of the investigated athletes with musculotendinous imbalances had a relative deficit in tendon stiffness (maximum tendon strain ≥ 9%). Considering the increased tendon injury risk associated with high-level tendon strain [22], this concept may be applied to prevent tendon overuse injuries, especially in populations with a high prevalence of tendinopathies and muscle–tendon imbalances.

## Data Availability

The datasets used and/or analyzed during the current study are available from the corresponding author on reasonable request.

## Abbreviations

M1 … 4: Measurement 1 … 4
MVC: Maximum voluntary contraction
PSF: Peak spatial frequency
VISA-P: Victorian institute of sports assessment–patellar
